# *GSTP1* polymorphism predicts treatment outcome and toxicities for breast cancer

**DOI:** 10.18632/oncotarget.18513

**Published:** 2017-06-16

**Authors:** Jie Ma, Shao-Liang Zhu, Yang Liu, Xiang-Yang Huang, Dan-Ke Su

**Affiliations:** ^1^ Department of Radiology, Affiliated Tumor Hospital of Guangxi Medical University, Nanning, China; ^2^ Department of Hepatobiliary Surgery, Affiliated Tumor Hospital of Guangxi Medical University, Nanning, China; ^3^ Department of Radiotherapy, Affiliated Tumor Hospital of Guangxi Medical University, Nanning, China

**Keywords:** GSTP1, breast cancer, treatment outcome, toxicities, meta-analysis

## Abstract

This study aimed to investigate the association of the *GSTP1* gene polymorphism with the outcomes and toxicities of treatments in breast cancer. Odds ratios (ORs) and 95% confidence intervals (95% CIs) were calculated for the association of *GSTP1* polymorphism with tumour response and toxicities, and the hazard ratios (HRs) and 95% CIs were calculated for the association between *GSTP1* polymorphism and overall survival (OS). The statistical analysis showed that the *GSTP1* polymorphism was not associated with tumour response or OS. A significant increase in the incidence of toxicities was observed (GA *vs*. AA OR = 1.45, 95% CI = 1.04–2.01, *P* = 0.028; GG *vs*. AA OR = 1.47, 95% CI = 1.03–2.10, *P* = 0.036; recessive model OR = 1.54, 95% CI = 1.13–2.09, *P* = 0.006; and allele model OR = 1.35, 95% CI = 1.07–1.71, *P* = 0.011), especially in the chemotherapy ± surgery group (GA *vs*. AA OR = 1.64, 95% CI = 1.05–2.56, *P* = 0.030; recessive model OR = 1.72, 95% CI = 1.17–2.54, *P* = 0.006; and allele model OR = 1.57, 95% CI = 1.11–2.21, *P* = 0.010). Our results indicate that the *GSTP1* polymorphism may be associated with increased toxicity, especially in patients treated with chemotherapy ± surgery.

## INTRODUCTION

Breast cancer in 2012 was both the most common cancer worldwide, with 1.7 million new cases, and the leading cause of cancer-related deaths in women [1]. Breast cancer is commonly treated with chemotherapy either as an adjuvant systemic treatment after primary surgery or as neoadjuvant therapy before surgery. The neoadjuvant treatment attempts to reduce the tumour stage, with the goal of surgically resecting the mass. Currently, radiotherapy is commonly used after primary surgery to reduce the risk of recurrence [2]. However, patient response to treatment with chemotherapy and radiotherapy is quite variable [3–5]. Inter-patient variations in clinicopathologic characteristics such as clinical disease stage, lymph node status, and hormone receptor expression, could have a large influence on treatment outcomes. Increasing evidence suggests that drug-metabolizing enzymes may play an important role in inter-patient variations, which could affect treatment response and toxicities [6, 7].

Glutathione S-transferases (GSTs) are a superfamily of phase-II metabolic enzymes that play a key role in cellular resistance mechanisms [8]. GSTs detoxify cytotoxic agents by catalysing the reduction of these compounds through their conjugation with glutathione [9]. The *GSTP1* gene, a member of the *GST* family, is located on chromosome 11q13, which contains 7 exons and 6 introns. Genetic polymorphisms involving an adenine to guanine transition (rs1695) at codon 105 in exon 5 of the *GSTP1* gene results in amino acid substitution from isoleucine to valine (Ile→Val). This substitution decreases the enzymatic activity of glutathione-S-transferase P1 (GSTP1) and alters the pharmacokinetics of cyclophosphamide, which may influence treatment outcomes and toxicity for breast cancer [10].

Studies have investigated the associations of the *GSTP1* (A313G) gene polymorphism with treatment response, prognosis, and toxicities for breast cancer [11–41]. However, these findings failed to reach a consensus owing to a lack of data and inconsistencies in the results between these studies. Therefore, a systematic review and meta-analysis was conducted to evaluate the influence of the *GSTP1* (A313G) polymorphism on treatment outcomes and toxicities in patients with breast cancer.

## MATERIALS AND METHODS

### Literature search and inclusion criteria

PubMed, EMBASE, Cochrane Library, and China National Knowledge Infrastructure were searched for relevant studies up to August 29th, 2016, by using the following terms: “glutathione S-transferase,” “glutathione S-transferase P1,” “*GSTP1*,” “breast cancer,” “breast carcinoma,” and “breast neoplasm.” Studies were manually filtered without language restrictions. Additional studies were identified by screening references and relevant reviews.

Studies were included if they met the following criteria: (1) inclusion of patients who were treated for breast cancer; (2) evaluation of associations between *GSTP1* and treatment outcomes, as well as toxicities after radiotherapy and/or chemotherapy; (3) treatment outcomes including tumour response and overall survival (OS), with toxicities including all adverse effects; and (4) provision of adequate data for calculation of both odds ratios (ORs) and hazard ratios (HRs) with 95% confidence intervals (CIs).

### Data extraction

The following items were gathered independently from all eligible studies by two investigators (JM and YL): first author's name, year of publication, country, number of patients, genotyping methods, median follow-up, treatment protocols, treatment outcomes, and toxicities. Any disagreements were resolved through discussion and consensus.

### Risk of bias

The risk of bias was assessed by reviewers independently using a modified Ottawa classification for observational studies [42]. Any disagreements were resolved by consensus.

### Statistical analysis

The responses were estimated according to Response Evaluation Criteria in Solid Tumors, including complete response (CR), partial response (PR), stable disease (SD), and progressive disease (PD). Patients with CR and PR were categorized as the responder group, and patients with SD and PD were categorized as the non-responder group. Toxicities were defined as all adverse effects that occurred after treatment with chemotherapy and/or radiotherapy. ORs with 95% CIs were used to evaluate the association between *GSTP1* and tumour response and toxicities based on raw data. Cox proportional HRs and 95% CIs for OS were also calculated using the most adjusted HR in each study. In this meta-analysis, we examined the association of variant genotypes of *GSTP1* polymorphism with treatment outcome and toxicities.

The heterogeneity was assessed using the *Q* test with a significance level of *P* < 0.05. The *I*^2^ statistic was used to test the heterogeneity among the included studies [43]. A fixed-effect model (Mantel-Haenszel method) was applied if heterogeneity was not significant [44]. Otherwise, a random effect model (DerSimonian and Laird method) was utilized [45].

Subgroup analysis was carried out based on ethnicity, sample size, and therapeutic method. Ethnic subgroups consisted of three groups: East Asian (Chinese and Japanese), South Asian (Indian and Bangladeshi), and mixed descent (American, Canadian, and Brazilian). Sample size was divided into a large group (≥ 100 cases) and a small group (< 100 cases). Therapeutic methods included chemotherapy ± surgery, radiotherapy ± surgery, and chemotherapy + radiotherapy ± surgery. Potential publication bias was assessed using a Funnel plot [46] and Egger's test [47]. Statistical analyses were conducted with STATA version 11.0 (Stata Corporation, College Station, Texas, USA). All *P* values were 2-sided and *P* < 0.05 was considered statistically significant.

## RESULTS

### Study characteristics

**Figure 1:**
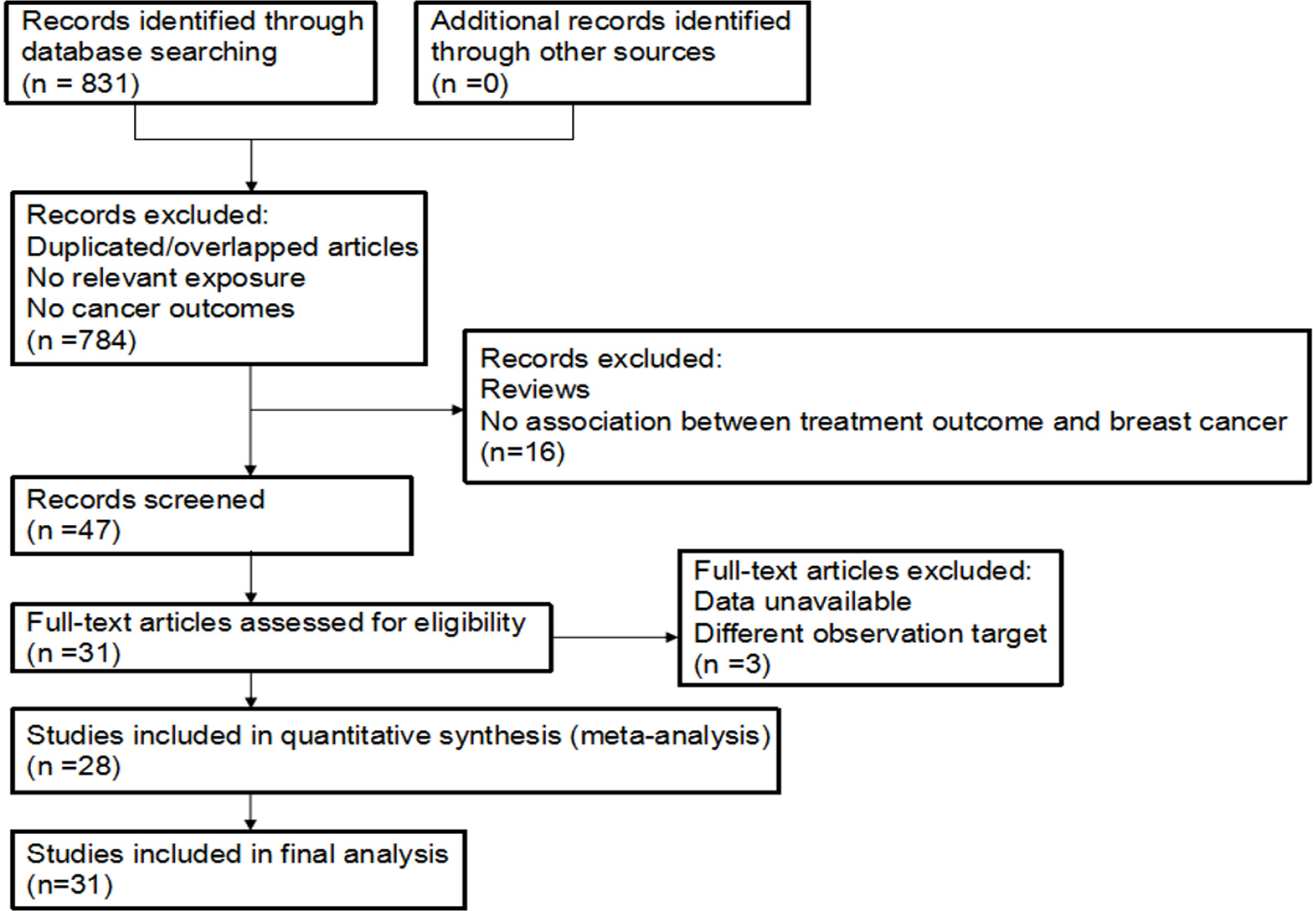
The flowchart of this meta-analysis

A total of 831 potentially relevant publications were systematically identified. Of them, 784 studies were excluded because they were reviews, letters, comments, or irrelevant studies. An additional study was excluded because data were not provided, and the authors could not be reached [11]. Another study was excluded because it regarded progression-free survival as an observation endpoint [12]. One study was excluded because it regarded CR + PR + SD as the responder group [13]. Another study was excluded owing to inclusion of familial breast cancer and sporadic breast cancer patients [14]. Furthermore, as one study used three different regimens of chemotherapy, it was treated as three articles [15]. After applying the exclusion criteria, 31 studies with a total of 7506 patients were included [14–41]. The study selection flowchart is summarized in Figure 1. The basic characteristics of all included studies are listed in Table 1. Sample sizes ranged from 40 to 1034 patients. Among the studies analysed, 15 reported tumour response events [15, 20–23, 26, 27, 31–35, 37, 38, 41], 13 reported OS [14, 17, 19, 23, 28, 29, 31–34, 38, 41], and 12 reported toxicities [16, 18, 24, 26, 27, 29, 30, 35–37, 39, 40]. Table 2 shows the quality indicators of the included studies.

**Table 1:** Characteristics of all included studies in this meta-analysis

Study	Country	Publication	Genotyping method	Number of patient	Treatment	toxicities	Median follow-up	specimen
Sweeney, et al. [17]	America	2000	PCR-RFLP	240	chemotherapy + radiotherapy, chemotherapy, radiotherapy	NP	58 months	tissue
Yang, et al. [29]	China	2005	Multiplex PCR	1034	chemotherapy	NP	5.3 years	blood
Ambrosone, et al. [16]	German	2006	Multiplex PCR	446	radiotherapy after surgery	skin toxicities	NP	blood
Zárate, et al. [39]	Spain	2007	PCR-RFLP	94	chemotherapy	haematological and non-haematological	NP	blood
Syamala, et al. [14]	India	2008	PCR	347	NP	NP	NP	blood
Kuptsova, et al. [18]	German	2008	Multiplex PCR	390	radiotherapy after surgery	telangiectasia	4.1 years	blood
Bewick, et al. [19]	Canada	2008	PCR	95	chemotherapy	NP	10.4 months	blood or bone marrow
Tang, et al. [20]	China	2009	PCR	126	chemotherapy	NP	6 weeks	blood
Oliveira, et al. [21]	Brazil	2010	PCR-RFLP	40	chemotherapy	NP	NP	blood
Yao, et al. [40]	America	2010	PCR-RFLP	458	chemotherapy	hematologic	10.8 years	tumor cell
Zhong, et al. [22]	China	2010	PCR	132	chemotherapy	NP	9 weeks	blood
Zhang (1), et al. [37]	China	2011	PCR-RFLP	120	chemotherapy	hematologic	NP	blood
Bai, et al. [23]	China	2012	PCR-RFLP	159	chemotherapy	NP	4 years	blood
Terrazzino, et al. [24]	Italy	2012	PCR	237	radiotherapy	skin fibrosis	63 days	blood
Raabe, et al. [25]	German	2012	PCR-RFLP	83	radiotherapy	erythema	NP	blood
Ji, et al. [26]	China	2012	PCR	153	chemotherapy	neutropenia	51 months	blood
Tulsyan, et al. [27]	India	2013	PCR-RFLP	100	chemotherapy	hematologic	NP	blood
Duggan, et al. [28]	America	2013	PCR	533	surgery, surgery and radiotherapy, and chemotherapy	NP	11.29 years	blood
Zhang (2), et al. [38]	China	2013	PCR-CTPP	219	chemotherapy	NP	4 years	blood
Zhao, et al. [15]	China	2014	PCR	252	chemotherapy	hematologic	NP	blood
Sugishita, et al. [30]	Japan	2014	PCR	102	chemotherapy	hematologic	967 days	blood
Liu, et al. [34]	China	2014	PCR	382	chemotherapy after surgery	NP	NP	blood
Zhou, et al. [31]	China	2015	PCR	420	chemotherapy after surgery	NP	5 years	blood
Wang (1), et al. [32]	China	2015	PCR-RFLP	310	chemotherapy	NP	5 years	blood
Wang (2), et al. [33]	China	2015	PCR-RFLP	262	chemotherapy	NP	NP	blood
Islam, et al. [35]	Bangladesh	2015	PCR-RFLP	256	chemotherapy	hematologic	NP	blood
Eckhoff, et al. [36]	Denmark	2015	PCR	150	chemotherapy	docetaxel-induced peripheral neuropathy	7.5 months	blood
Yuan, et al. [41]	China	2015	PCR-RFLP	273	chemotherapy	NP	5 years	blood

*PCR* polymerase chain reaction, *NP* not provided

**Table 2:** Quality assessment (risk of bias) of the included studies

Study	Ascertainment of outcome	Adjusting for confounders	Attrition bias	Patient selection
Sweeney, et al. [17]	Main confounders and any additional confounders	Yes	No risk	Consecutive
Yang, et al. [29]	Main confounders and any additional confounders	Yes	No risk	Consecutive
Ambrosone, et al. [16]	Main confounders and any additional confounders	No	No risk	Consecutive
Zárate, et al. [39]	Main confounders and any additional confounders	No	No risk	Selected/non-consecutive patients
Syamala, et al. [14]	Main confounders and any additional confounders	No	Unclear reporting	Selected/non-consecutive patients
Kuptsova, et al. [18]	Main confounders and any additional confounders	No	No risk	Consecutive
Bewick, et al. [19]	Main confounders and any additional confounders	Yes	No risk	Selected/non-consecutive patients
Tang, et al. [20]	Main confounders and any additional confounders	No	Unclear reporting	Consecutive
Oliveira, et al. [21]	Main confounders and any additional confounders	No	Unclear reporting	Consecutive
Yao, et al. [40]	Main confounders and any additional confounders	No	No risk	Consecutive
Zhong, et al. [22]	Main confounders and any additional confounders	No	Unclear reporting	Consecutive
Zhang (1), et al. [37]	Main confounders and any additional confounders	No	Unclear reporting	Selected/non-consecutive patients
Bai, et al. [23]	Main confounders and any additional confounders	Yes	No risk	Consecutive
Terrazzino, et al. [24]	Main confounders and any additional confounders	No	No risk	Consecutive
Raabe, et al. [25]	Main confounders and any additional confounders	No	Unclear reporting	Consecutive
Ji, et al. [26]	Main confounders and any additional confounders	No	Unclear reporting	Selected/non-consecutive patients
Tulsyan, et al. [27]	Main confounders and any additional confounders	No	Unclear reporting	Consecutive
Duggan, et al. [28]	Main confounders and any additional confounders	Yes	No risk	Consecutive
Zhang (2), et al. [38]	Main confounders and any additional confounders	Yes	No risk	Consecutive
Zhao, et al. [15]	Main confounders and any additional confounders	No	Unclear reporting	Consecutive
Sugishita, et al. [30]	Main confounders and any additional confounders	No	Unclear reporting	Selected/non-consecutive patients
Liu, et al. [34]	Main confounders and any additional confounders	Yes	No risk	Consecutive
Zhou, et al. [31]	Main confounders and any additional confounders	Yes	No risk	Consecutive
Wang (1), et al. [32]	Main confounders and any additional confounders	Yes	No risk	Consecutive
Wang (2), et al. [33]	Main confounders and any additional confounders	No	No risk	Selected/non-consecutive patients
Islam, et al. [35]	Main confounders and any additional confounders	No	Unclear reporting	Consecutive
Eckhoff, et al. [36]	Main confounders and any additional confounders	No	Unclear reporting	Consecutive
Yuan, et al. [41]	Main confounders and any additional confounders	Yes	No risk	Consecutive

### Quantitative synthesis

Tables 3 and 4 summarize the meta-analyses of the association of the *GSTP1* polymorphism with tumour response, OS, and toxicities, respectively. The meta-analysis was conducted using a fixed-effect model when *P* > 0.05 for the Q test, which indicated a lack of heterogeneity among studies; otherwise, a random-effect model was used.

**Table 3:** Summary of results in the association of GSTP1 polymorphism with tumor response and overall survival

GSTP1 Genotype			No. of studies	Tumor response					No. of studies	Overall survival				
				No. of patients	OR (95 % CI)	*P* value	*P* of heterogeneity	*I*^2^ (%)		No. of patients	HR (95 % CI)	*P* value	*P* of heterogeneity	*I*^2^ (%)
GA vs. AA	Overall		15	2941	1.32 (0.97–1.80)	0.073	0.001	63.2	13	4274	1.14 (0.97–1.33)	0.106	0.328	11.7
	Ethnicity	East Asian	12	2684	1.38 (0.99–1.92)	0.058	0.001	64.6	8	3059	1.10 (0.90–1.33)	0.347	0.289	17.9
		South Asian	2	217	1.43 (0.46–4.43)	0.533	0.054	73.1	2	347	1.53 (0.78–2.99)	0.218	0.739	0.0
		Mixed descent	1	40	0.38 (0.10–1.15)	0.158	NA	NA	3	868	1.19 (0.78–1.81)	0.423	0.132	50.6
	Sample size	Large	14	2901	1.38 (1.02–1.88)	0.038	0.001	63.1	12	4179	1.11 (0.94–1.31)	0.231	0.306	14.1
		Small	1	40	0.38 (0.10–1.45)	0.143	NA	NA	1	95	1.37 (0.88–2.13)	0.163	NA	NA
	Overall		14	2901	1.29 (0.79–2.13)	0.312	0.000	71.6	12	4149	0.94 (0.56–1.57)	0.814	0.000	80.3
	Ethnicity	East Asian	12	2684	1.19 (0.69–2.03)	0.531	0.000	73.7	8	3059	1.08 (0.57–2.06)	0.806	0.000	85.7
GG vs. AA		South Asian	2	217	2.41 (0.88–6.58)	0.086	0.467	0.0	1	222	0.30 (0.03–2.99)	0.305	NA	NA
		Mixed descent	NA	NA	NA	NA	NA	NA	3	868	0.72 (0.26–2.00)	0.525	0.052	66.2
GA + GG vs. AA (dominant model)	Sample size	Large	14	2901	1.29 (0.79–2.13)	0.312	0.000	71.6	11	4054	0.89 (0.51–1.57)	0.692	0.000	81.6
		Small	NA	NA	NA	NA	NA	NA	1	95	1.51 (0.75–3.02)	0.246	NA	NA
	Overall		15	2941	1.37 (0.97–1.94)	0.074	0.000	76.3	3	1048	1.74 (1.32–2.30)	< 0.001	0.140	49.2
	Ethnicity	East Asian	12	2684	1.43 (0.97–2.10)	0.068	0.000	78.6	1	420	2.53 (1.60–4.03)	NA	NA	NA
		South Asian	2	217	1.58 (0.55–4.56)	0.399	0.056	72.6	NA	NA	NA	NA	NA	NA
		Mixed descent	1	40	0.38 (0.10–1.15)	0.158	NA	NA	2	628	1.41 (0.99–2.00)	0.053	0.956	0.0
	Sample size	Large	14	2901	1.45 (1.02–2.06)	0.040	0.000	77.0	2	953	1.98 (1.14–3.45)	0.015	0.152	51.2
		Small	1	40	0.38 (0.10–1.45)	0.143	NA	NA	1	95	1.40 (0.92–2.12)	0.100	NA	NA
GG vs. AA+GA (recessive model)	Overall		14	2901	1.05 (0.70–1.57)	0.829	0.000	66.2	NA	NA	NA	NA	NA	NA
	Ethnicity	East Asian	12	2684	0.97 (0.63–1.48)	0.872	0.000	68.7	NA	NA	NA	NA	NA	NA
		South Asian	2	217	2.07 (0.78–5.47)	0.143	0.797	0	NA	NA	NA	NA	NA	NA
		Mixed descent	NA	NA	NA	NA	NA	NA	NA	NA	NA	NA	NA	NA
	Sample size	Large	14	2901	1.05 (0.70–1.57)	0.829	0.000	66.2	NA	NA	NA	NA	NA	NA
		Small	NA	NA	NA	NA	NA	NA	NA	NA	NA	NA	NA	NA
Allele model (G vs. A)	Overall		15	2941	1.26 (0.93–1.70)	0.134	0.000	82.2	2	601	1.32 (0.47–3.74)	0.601	0.004	87.7
	Ethnicity	East Asian	12	2684	1.28 (0.91–1.79)	0.156	0.000	84.6	2	601	1.32 (0.47–3.74)	0.601	0.004	87.7
		South Asian	2	217	1.54 (0.76–3.10)	0.231	0.101	62.9	NA	NA	NA	NA	NA	NA
		Mixed descent	1	40	0.38 (0.10–1.45)	0.143	NA	NA	NA	NA	NA	NA	NA	NA
	Sample size	Large	14	2901	1.31 (0.96–1.78)	0.089	0.000	83.1	2	601	1.32 (0.47–3.74)	0.601	0.004	87.7
		Small	1	40	0.38 (0.10–1.45)	0.143	NA	NA	NA	NA	NA	NA	NA	NA

*GSTP1* glutathione S-transferase P1, OR odds ratio, CI confidence interval, HR hazard ratio, NA not available.

**Table 4:** Summary of results in the association of *GSTP1* polymorphism with toxicities

GSTP1 Genotype			No. of studies	toxicities				
				No. of patients	OR (95 % CI)	*P* value	*P* of heterogeneity	*I*^2^ (%)
GA vs. AA		Overall	11	1950	**1.45 (1.04–2.01)**	0.028	0.024	51.5
	Therapeutic method	Chemotherapy ± surgery	8	1031	**1.64 (1.05–2.56)**	0.030	0.032	54.2
		Radiotherapy ± surgery	3	919	1.10 (0.79–1.52)	0.579	0.144	48.5
GG vs. AA		Overall	11	1950	**1.47 (1.03–2.10)**	0.036	0.709	0.0
	Therapeutic method	Chemotherapy ± surgery	8	1031	1.58 (0.98–2.55)	0.059	0.776	0.0
		Radiotherapy ± surgery	3	919	1.33 (0.77–2.28)	0.306	0.207	36.4
GA + GG vs. AA (dominant model)		Overall	14	2747	1.35 (0.99–1.83)	0.058	0.001	61.3
	Therapeutic method	Chemotherapy ± surgery	10	1591	1.40 (0.90–2.18)	0.133	0.001	68.8
		Radiotherapy ± surgery	4	1156	1.24 (0.93–1.65)	0.143	0.213	33.3
GG vs. AA+GA (recessive model)		Overall	12	2044	**1.54 (1.13–2.09)**	0.006	0.330	11.8
	Therapeutic method	Chemotherapy ± surgery	9	1125	**1.72 (1.17–2.54)**	0.006	0.526	0.0
		Radiotherapy ± surgery	3	919	1.12 (0.47–2.67)	0.792	0.092	58.2
G vs. A (Allele model)		Overall	11	1950	**1.35 (1.07–1.71)**	0.011	0.023	51.6
	Therapeutic method	Chemotherapy ± surgery	8	1031	**1.57 (1.11–2.21)**	0.010	0.013	60.7
		Radiotherapy ± surgery	3	919	1.12 (0.89–1.40)	0.346	0.568	0.0

*GSTP1* glutathione S-transferase P1, OR odds ratio, CI confidence interval, NA not available.

### Tumor response

**Figure 2:**
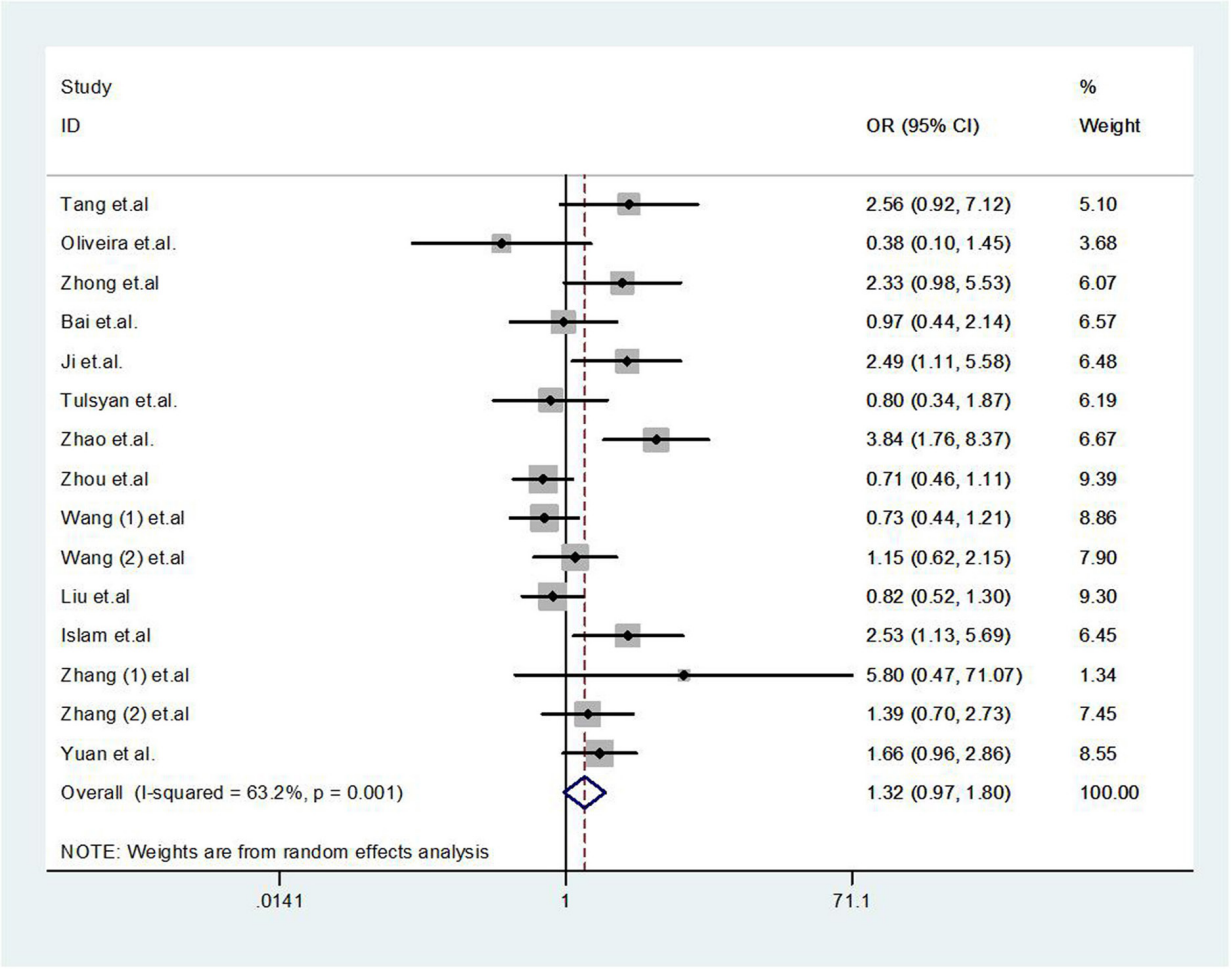
Forest plot of tumor response for *GSTP1* gene polymorphism in breast cancer patients (GA *vs*. AA)

**Figure 3:**
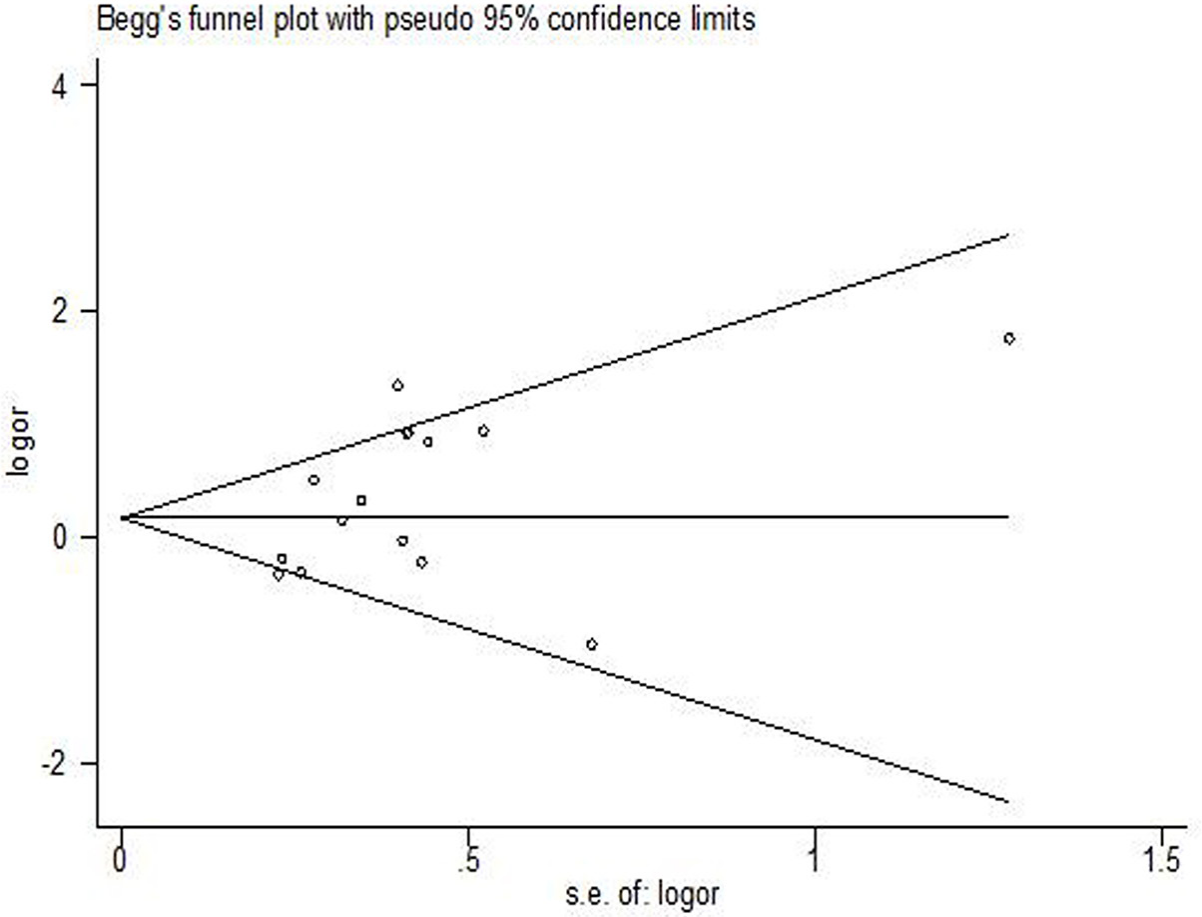
Funnel plot of *GSTP1* gene polymorphism for assessment of publication bias: tumor response (GA *vs*. AA)

There was no significant association between the *GSTP1* polymorphism and tumour response (GA *vs.* AA OR = 1.32, 95% CI 0.97–1.80, *P* = 0.073, Figure 2; GG *vs.* AA OR = 1.29, 95% CI 0.79–2.13, *P* = 0.312; dominant model OR = 1.37, 95% CI 0.97–1.94, *P* = 0.074; recessive model OR = 1.05, 95% CI 0.70–1.57, *P* = 0.829; or allele model OR = 1.26, 95% CI 0.93–1.70, *P* = 0.134). Publication bias was observed in the Funnel plot and Egger's test (GG *vs.* AA, *P* = 0.023; and recessive model, *P* = 0.034), but not for other models (GA *vs.* AA, *P* = 0.066, Figure 3; dominant model *P* = 0.052, or allele model *P* = 0.054).

### Overall survival

The *GSTP1* polymorphism was associated with OS in the dominant genetic model (HR = 1.74, 95% CI 1.32–2.30, *P <* 0.001), but not other genetic models (GA *vs.* AA, HR = 1.14, 95% CI 0.97–1.33, *P* = 0.106; GG *vs.* AA, HR = 0.94, 95% CI 0.56–1.57, *P* = 0.814; or allele model HR = 1.32, 95 % CI 0.47–3.74, *P* = 0.601), as shown in Table 3. Publication bias was not observed in the Funnel plot or Egger's test (GA *vs.* AA, *P* = 0.365; and GG *vs.* AA, *P* = 0.719).

### Toxicities

**Figure 4:**
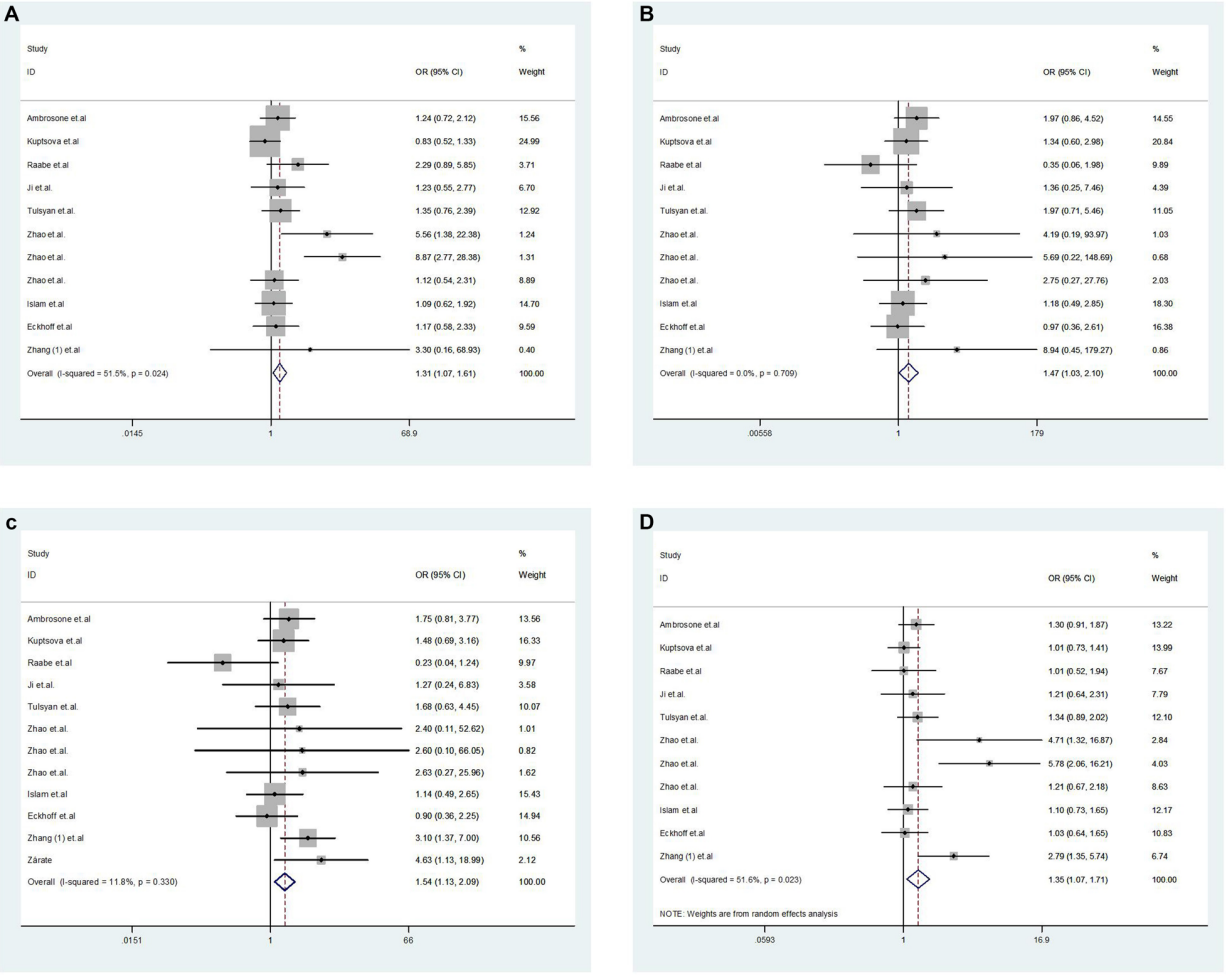
Forest plot of toxicities for *GSTP1* gene polymorphism in breast cancer patients ((**A)**: GA *vs.* AA; (**B**): GG *vs.* AA; (**C**): recessive model; (**D**): allele model).

Pooled results showed that there was a significant increase of toxicities (GA *vs.* AA OR = 1.45, 95% CI 1.04–2.01, *P* = 0.028, Figure 4A; GG *vs.* AA OR = 1.47, 95% CI 1.03–2.10, *P* = 0.036, Figure 4B; recessive model OR = 1.54, 95% CI 1.13–2.09, *P* = 0.006, Figure 4C; and allele model OR = 1.35, 95% CI 1.07–1.71, *P* = 0.011, Figure 4D; Table 4). The dominant model was not found to be significantly associated with toxicity (OR = 1.35, 95% CI 0.99–1.83, *P* = 0.058). Publication bias was observed in the Funnel plot and Egger's test for the genetic models (GA *vs.* AA, *P* = 0.008; dominant model, *P* = 0.011; and allele model, *P* = 0.008) There was no publication bias in other models (GG *vs.* AA, *P* = 0.271; and the recessive model, *P* = 0.957).

### Subgroup analysis

In subgroup analyses, the *GSTP1* polymorphism was associated with increased tumour response when the sample size was large (GA *vs.* AA OR = 1.38, 95% CI 1.02–1.88, *P* = 0.038; and dominant model OR = 1.45, 95% CI 1.02–2.06, *P* = 0.829), but this association was not found when the sample size was small, or with any ethnicity subgroup. In addition, no associations between *GSTP1* polymorphism and OS were found in either large or small sample sizes. The estimated results showed that there was an increased incidence of toxicities after chemotherapy ± surgery in three genetic models: GA *vs.* AA (OR = 1.64, 95% CI 1.05–2.56, *P* = 0.030), recessive model (OR = 1.72, 95% CI 1.17–2.54, *P* = 0.006), and allele model (OR = 1.57, 95% CI 1.11–2.21, *P* = 0.010; Table 4). However, we failed to find such an association in the radiotherapy ± surgery group.

## DISCUSSION

While the breast cancer treatment response of chemotherapy and/or radiotherapy cannot be optimally predicted, the consequence of gene polymorphism affecting drug efficacy, through encoding metabolizing enzymes and drug transporters, has been confirmed [48]. For breast cancer, anthracycline/paclitaxel-based agents are often effective, which is due to DNA damage, as well as mitochondrial membrane disruption, triggering the apoptotic mechanism and contributing to tumour cell death by the generation of reactive oxygen species (ROS). GSTs (particularly GSTP1) are multifunctional enzymes involved in the protection of cellular components targeted by anticancer drugs. GSTs detoxify chemotherapeutic drugs, or their metabolites, by catalysing the reduction of these compounds through conjugation with glutathione [49]. Therefore, this function of GSTs may result in tumour resistance to cytotoxic agents during chemotherapy [50]. However, the substitution of Ile to Val at codon 105 would result in the decrease of this function, and thus potentially cause an increase in the efficacy of chemotherapy [10]. Previous studies have investigated the association between the *GSTP1* polymorphism and treatment outcomes in other cancers [52–53]. In these studies, no significant association between the *GSTP1* polymorphism and tumour response from platinum-based chemotherapy was found in either colorectal cancer or gastric cancer [51, 52]. On the contrary, the variant G *allele* was significantly associated with positive response to platinum-based chemotherapy in East-Asian patients with non-small cell lung cancer [53]. A significantly longer OS was observed in GG + GA genotypes than in AA genotypes in gastric cancer [52]. In the current meta-analysis, there was no significant association between the *GSTP1* polymorphism and tumour response. With these inconsistent results, additional studies using uniform evaluation standards are needed in future to sufficiently determine the association between the *GSTP1* polymorphism and tumour response.

Chemotherapy and radiotherapy can potentially be interrupted by treatment toxicities. Radiotherapy often has short-term toxicities such as skin erythema and irritation, as well as medium- and long-term toxicities such as breast oedema, pain, fibrosis, and/or telangiectasia. Chemotherapy is usually accompanied by toxicities, such as hematologic, cardiac, and hepatic dysfunction, and vomiting. Severe toxicities might result in treatment interruption and thereby affect treatment efficacy. The *GSTP1* Ile to Val substitution has been associated with reduced enzyme activity in the removal of chemotherapy agents [3]. Therefore, it may lead to several toxicities during chemotherapy. Two previous studies have evaluated the association between the *GSTP1* polymorphism and toxicities. Both studies found that patients with the AA genotype were at significantly higher risk of haematological and neurological toxicities, compared with patients expressing the AG or GG genotypes [54, 55]. In the current study, we found that the G*STP1* polymorphism was associated with increased toxicities, especially in patients treated with chemotherapy ± surgery. However, this function is advantageous for decreasing toxicities of patients receiving radiotherapy ± surgery. As radiation results in the generation of ROS and lipid peroxidation, nuclear *GSTP1* plays a direct role in the cellular sensitivity to oxidative stress. This oxidative stress is caused by hydrogen peroxide through the formation of lipid-peroxide modified DNA. In this meta-analysis, no association was observed between the *GSTP1* polymorphism and toxicities due to radiotherapy ± surgery.

Although an effort was made to conduct an accurate and comprehensive analysis, this study has several limitations. First, some factors that may lead to heterogeneity and thus have an influence on treatment outcome—such as treatment options (adjuvant and/or neoadjuvant chemotherapy), chemotherapeutic agents, breast cancer subtypes (hormone receptor-positive, Her2-positive, and triple-negative), and TNM staging status—were not strictly described in some studies. Therefore, these factors could not be stratified into subgroups with great detail. Furthermore, the major sources of heterogeneity where not detected, except for subgroup analysis by ethnicity, sample size, and therapeutic method. Second, publication bias existed in this meta-analysis. This may be due to the absence of some negative trials, which may lead to overestimate the treatment effects.

In conclusion, our meta-analysis suggests that *GSTP1* polymorphism may be associated with increased incidence of toxicities, especially in patients treated with chemotherapy ± surgery. Nevertheless, no significant associations were found between the *GSTP1* polymorphism and tumour response or OS.
